# Economy of Labour in Hospital Management

**Published:** 1912-07-27

**Authors:** W. Fenwick

**Affiliations:** Assistant Secretary, Hull Royal Infirmary.


### "ECONOMY OF LABOUR IN HOSPITAL MANAGEMENT."

### An Assistant Secretary's Further Suggestions.

By W. FENWICK, Assistant Secretary, Hull Royal Infirmary.

Following on Mr. Alfred M. Hooper's article of a fortnight ago on "Economy of Labour in Hospital Management" regarding the use of printed forms intended to facilitate the work of the hospital office staff, I propose to supplement the methods explained by the following. My first concerns the cash counterfoil receipt book. I am not aware what form of receipt is in general use at charitable institutions, but where the system obtains of giving a temporary receipt, and this to be later followed by the treasurer's official receipt, I am confident the system as set forth in this article will be welcomed, saving, as it does, a great amount of labour and some expense.

I will first outline the method once in force at the institution I am connected with, and then explain the present manner of receipting amounts contributed.

The temporary receipt book used by collectors contained a double perforated counterfoil, in addition, of course, to the receipt proper. The use of same, therefore, necessitated three distinct entries for each item of cash received. On cash being handed over to the secretary each morning the collectors also passed their day's counterfoils, retaining corresponding ones as a means of reference.

The secretary's clerk then made out the official receipts, which, together with collectors' counterfoils, were submitted to the treasurer for scrutiny and signature, the receipts being afterwards forwarded direct to the subscribers.

The operations involved were as follows: Collector, three entries. Secretary's staff, making out official receipts and counterfoils, placing with each the collector's counterfoil, addressing same after obtaining treasurer's signature, and, in addition to treasurer's time, there was the cost of postage and three sets of official receipt books for various contributions. The time spent in connection with this system was considerable. In place of this is now used a carbon receipt book, which does away with three-quarters of the above labour, and, which is equally important, is a safe check on all cash received. The points assuring this safety and approved by auditors are these: (1) Double-sided carbon paper used. (2) Receipt receiving carbon impress given to contributors, the top or original being retained. (3) All receipts to be clear, errors necessitating fresh ones being written. (4) Before tearing out receipt the payer is requested to examine and initial same, and a circle mark is provided for this purpose. About this circle mark the following note appears: "To provide an efficient check on amounts paid to this charity, contributors are respectfully requested to initial collector's counterfoil, the carbon impress of which should appear in circle on your receipt. Where alteration in amount is necessary collectors have instructions to cancel and provide a clear receipt." Each receipt also bears the perforation of the institution's stamp.

To simplify the work of sorting accounts for payment in order required and in accordance with the uniform system, type the various items of expenditure on cards, using a separate card for each department, and headed: (1) Provisions. (2) Surgery and Dispensary. (3) Domestic. (4) Establishment, and so on.

By using these, accounts can be rapidly sorted by placing same under respective departmental cards and finally sorting into item order, taking one card at a time. This saves memorising the long list of expenditure items and ensures perfect order for subsequent entry into expenditure book.

This is a printed slip issued by the wards showing the condition of all patients, and enables the porter to answer enquiries without repeatedly troubling the wards. About six stages are indicated thereon, and opposite each stage patients' numbers are placed.

I think all will agree with the writer of the first article on this subject that a recognised set of forms would be of practical use to the secretary's department, but as to what extent adopted there is something to be said as in the previous note, p. 376, on "The Value of Printed Forms." Where a circular takes the place of a secretary's letter is an instance in point. There are methods necessary and methods unnecessary, but a little discrimination will separate one from the other.

